# APECED: A Paradigm of Complex Interactions between Genetic Background and Susceptibility Factors

**DOI:** 10.3389/fimmu.2013.00331

**Published:** 2013-10-23

**Authors:** Lucia De Martino, Donatella Capalbo, Nicola Improda, Federica D’Elia, Raffaella Di Mase, Roberta D’Assante, Ida D’Acunzo, Claudio Pignata, Mariacarolina Salerno

**Affiliations:** ^1^Pediatric Section, Department of Translational Medical Sciences, “Federico II” University, Naples, Italy

**Keywords:** autoimmune polyglandular syndrome type 1, APECED, autoimmune regulator gene, phenotypic variability, tolerance

## Abstract

Autoimmune polyendocrinopathy-candidiasis-ectodermal dystrophy (APECED) is a rare autosomal recessive disease, caused by mutations of a single gene named Autoimmune regulator gene (AIRE) which results in a failure of T-cell tolerance. Central tolerance takes place within the thymus and represents the mechanism by which potentially auto-reactive T-cells are eliminated through the negative selection process. The expression of tissue-specific antigens (TSAs) by medullary thymic epithelial cells (mTECs) in the thymus is a key process in the central tolerance and is driven by the protein encoded by AIRE gene, the transcription factor autoimmune regulator (AIRE). A failure in this process caused by AIRE mutations is thought to be responsible of the systemic autoimmune reactions of APECED. APECED is characterized by several autoimmune endocrine and non-endocrine manifestations and the phenotype is often complex. Although APECED is the paradigm of a monogenic autoimmune disorder, it is characterized by a wide variability of the clinical expression even between siblings with the same genotype, thus implying that additional mechanisms, other than the failure of Aire function, are involved in the pathogenesis of the disease. Unraveling open issues of the molecular basis of APECED, will help improve diagnosis, management, and therapeutical strategies of this complex disease.

## Introduction

Autoimmune Polyglandular Syndrome Type 1 (APS-1), also called Autoimmune polyendocrinopathy-candidiasis-ectodermal dystrophy (APECED), is a rare autosomal recessive disease caused by mutations of the autoimmune regulator gene (AIRE). Immunologically, APECED is characterized by destruction of the target organs by a cellular- and/or antibody-mediated attack (1). In the past decade, much interest has been focused on the pathogenesis of this syndrome. Indeed, APECED represents a paradigm of genetically determined systemic autoimmunity. However, the great variability that characterizes APECED, irrespectively of the AIRE genotype, implies that several factors are involved in the disease phenotypic expression.

In this review, we will focus on the complex pathogenesis of APECED and on the potential interfering factors involved in the clinical expression of the disease.

## The Basis of the Immunological Tolerance

Tolerance represents a state of immunologic non-responsiveness in the presence of a particular antigen. In this context, T-cell tolerance is crucial for the creation of a proper T-cell repertoire, able to respond to a huge number of foreign antigens, but preventing autoimmune reactions. Imposition and regulation of self-tolerance within the T-cell repertoire is exerted at two levels: (1) central tolerance (development and selection of T-cells in the thymus) and (2) peripheral tolerance (deletion, anergy of mature T-cells in lymphoid and non-lymphoid organs) (2).

**Figure 1:**
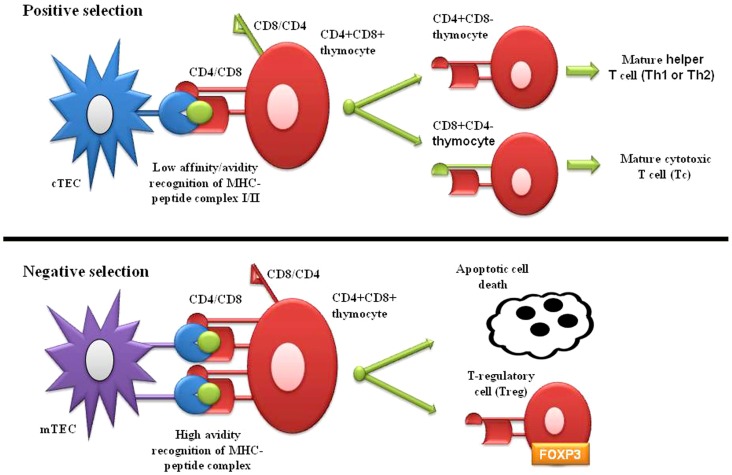
**Positive and negative selection of immature thymocytes within thymus**.

T-cell central tolerance, established within the thymus, mostly relies on two main mechanisms: negative selection, also referred to as clonal deletion of maturing thymocytes and positive selection of maturing T-cells able to bind to a surface major histocompatibility complex (MHC) molecule with mild threshold of reactivity (Figure 1). The thymus provides the necessary environment for thymopoiesis and establishment and maintenance of self-tolerance (3–5). Thymus contains thymic epithelial cells (TECs) that form a complex three-dimensional network organized in cortical and medullary compartments (6). On entering the thymus, immature thymocytes promote the differentiation of precursor thymic epithelial cells (pTECs) into cortical TECs (cTECs) and medullary TECs (mTECs), playing an important role in the formation of the thymic microenvironment (7–9). During postnatal life, hematopoietic progenitors enter the thymus from the bloodstream (10) and cells committed to the T lineage undergo division, mostly within the double-negative (DN) stage of the T-cell development. The first checkpoint is the rearrangement of T-cell receptor (TCR) β and α locus. Expression of αβ TCR heterodimers on the cell surface allows DN thymocytes to progress to the double-positive (DP) CD4+CD8+ stage. At DP stage, the TCR affinity for self-peptide-MHC on mTECs within the thymus determines thymocyte’s fate. mTECs express a wide array of tissue-specific antigens (TSAs) in the context of MHC class II molecules; these TSAs include self-proteins derived from different organs in the body. DP thymocytes expressing TCRs that do not bind self-peptide-MHC complexes are programed to undergo “death by neglect” or apoptosis. Only about 5% of DP has a low affinity for self-peptide-MHC complexes and differentiate to CD4+CD8− or CD4−CD8+ single positive (SP) lineage (positive selection) (11–13). DP thymocytes with high-affinity TCR for MHC complexes represent a potential reservoir of auto-reactive lymphocytes and “clonal deletion” (negative selection) is the main mechanism in the thymus to preserve self-tolerance (14, 15). Compelling evidence indicates that an altered promiscuous thymic expression of TSAs leads to autoimmunity. In the autoimmune attack, T helper cells (Th) escaped to self-tolerance, produce pro-inflammatory cytokines able to begin inflammation and activate auto-reactive B-cells, resulting in autoantibodies production, which lead to tissue inflammation and damage (1). Some of the thymocytes that recognize self-peptide-MHC complexes with high-affinity express Foxp3 and through “clonal diversion” mature as regulatory T-cells (Tregs), which are able to suppress auto-reactive T-cells in the periphery (16–18). The central tolerance is not able alone to completely remove mature T-cells with self-antigens specificity, therefore additional mechanisms in the periphery are also needed to maintain immunological tolerance.

The peripheral tolerance recognizes as possible mechanisms the induction of functional anergy, deletion of auto-reactive clones, and the suppressive action of T-regulatory cells (Tregs). Anergy is a state of long-term hyporesponsiveness with inactivation of self-reactive T-cells in the presence of a TCR signal but in the absence of a second costimulatory signal, necessary to T-cell activation. Deletion of self-reactive lymphocytes is achieved in both the thymus and the periphery by apoptosis through interaction of Fas/FasL. The function of Tregs (Foxp3-expressing CD4 T-cells) is to suppress immune responses through numerous mechanisms including the production of anti-inflammatory cytokines, direct cell–cell contact, and by modulating the activation state and function of antigen-presenting cell (APC) (19). An additional mechanism involved in controlling reactivity to self in the periphery is NK cell activity.

## AIRE and the Maintenance of Immunological Tolerance

**Figure 2:**
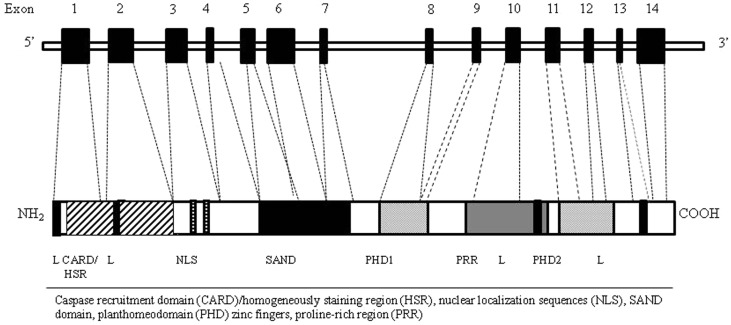
**AIRE gene (top) and corresponding protein (bottom) with functional domains**. The Aire protein is composed by nuclear localization sequences (NLS), two planthomeodomain (PHD) zinc fingers, a caspase recruitment domain (CARD)/homogeneously staining region (HSR), a SAND domain, and a proline-rich region (PRR).

Autoimmune regulator gene encodes for a transcription factor (Aire) involved in the maintenance of tolerance. In humans, the AIRE gene maps to chromosome 21q22.3 (20, 21). It consists of 14 exons spanning 11.9 kb of genomic DNA (22) and encodes a 545 amino acid protein with a molecular weight of 58 kDa that works as a “non-classical” transcriptional factor in immune-related organs. The highest level of AIRE expression has been detected within the thymus (23) in mTECs, followed by thymic dendritic cells (DCs). In addition to the thymus, low level of Aire seems to be expressed in secondary lymphoid organs, such as lymph nodes, fetal liver, and spleen (24, 25). The Aire protein, mostly localized in the cell nucleus, is composed by specific domains including the amino-terminal HSR domain, the nuclear localization signal (NLS), the Sp100, AIRE1, nucP41/75, DEAF 1 (SAND) domain, two plant homeodomain (PHD) type zinc fingers, and four LXXLL motifs (26) (Figure 2). The HSR region has been shown to be responsible for the dimerization of the polypeptides belonging to the Sp100 protein family (27). The SAND domain is important for AIRE transactivation capacity and subcellular localization. The PHD zinc fingers are often found in proteins involved in the regulation of transcription (28). The LXXLL motifs are found on coactivators nuclear receptors and proline-rich regions (PRR) and are also associated to transcription regulation (29). Although the precise molecular mechanism is still unclear, Aire seems to regulate the transcription process acting as a coactivator in a large transcriptional complex (30), and interacting with a large set of partners, divided into four main classes based on their function: nuclear transport, chromatin binding/structure, transcription, and pre-mRNA processing factors (31). The first protein reported to bind to AIRE was CREB-binding protein (CBP) (32). Its interaction with AIRE may lead to promotion of gene transcription through histone acetylation and the recruitment of chromatin-transcription factors (33). Other AIRE partners have been identified, such as DNA protein kinase (DNA-PK), SP-RING domain protein inhibitor of activated STAT1 (PIAS1), positive transcription elongation factor b (P-TEFb) (34–36). Moreover, it has been proposed a possible epigenetic control of the AIRE target genes since AIRE’s PHD1 finger domain appears to be able to bind histone three molecules with unmethylated lysine at position 4, generally associated with repressed genes (37). Overall, it is possible that Aire mediates the expression of TSA in mTECs through its co-transcriptional partners (38). The intriguing question is how the AIRE gene alone can influence the transcription of such a large number of TSA genes. Indeed, two models have been suggested to explain the action of Aire: transcription model and maturation model. In the transcription model, TSAs are considered to be the direct target genes of Aire’s transcriptional activity and the lack of Aire protein within the cell would result in the defective TSA gene expression, while the maturation program of mTECs would be in principle unaffected. The maturation model suggests that Aire may affect the thymic microenvironment more globally than through simple control of TSA expression levels. Consequently, in keeping with the latest model the regulation of TSA gene expression might not be the major defect of Aire-deficient mTECs responsible for impaired negative selection (39).

Although the exact role of AIRE in controlling T-cell tolerance is still largely unclear, several mechanisms have been suggested. Functional alterations of AIRE may affect processing and/or presentation of self-antigens within the mTECs (40). The process of thymocyte maturation (41), the attraction of mature thymocytes to their final location for a proper negative selection (40, 42), the control of cross-presentation through alteration of the relationship between APCs and mTECs (43) may also represent potential mechanisms by which AIRE alterations may lead to functional abnormalities of the central tolerance. The alteration in the balance between negative selection and regulatory T-cell production (44) may also be implicated in the pathogenesis. In addition, Aire may also play a role in the proper differentiation of the thymic medullary epithelium, in the induction of apoptosis in end-stage terminally differentiated mTECs (39) as well as in mTECs’ differentiation program. In particular, evidence suggests that lack of Aire in mTECs results in an arrest of the differentiation program, with the cells remaining at the premature stage just before terminal differentiation (45, 46).

## The Clinical Counterpart of AIRE Mutation: APECED

### Genetic background

Mutations in AIRE gene result in development of APECED, which represents the paradigm of a genetically determined failure of central tolerance leading to autoimmunity (46). APECED is a rare autoimmune syndrome, but it has been reported worldwide showing a relatively higher prevalence in genetically isolated populations such as Iranian Jews (1:9,000) (47), Finns (1:25,000) (48, 49), and Sardinians (1:14,400) (50). It is also quite frequent in Norway (1:90,000) (51) and in some regions of Italy (52–55). The most frequent model of inheritance is autosomal recessive, even though a dominant pattern has also been sporadically reported (56). So far, over 70 different mutations of AIRE have been documented (2). Due to the molecular organization and the complexity of intermolecular connection of Aire, it would be expected that different mutation in the molecule might imply different functional abnormalities, thus being associated with a variable phenotypic expression. Single nucleotide substitutions, small insertions, deletions, and mutations affecting splice consensus sequences have been identified along the entire coding region, and include either nonsense or frameshift mutations that result in truncated polypeptides, or missense mutations that result in single amino acid-changing (27). Most of these AIRE mutations lead to a change in its subcellular location altering the distribution of the protein between the nucleus and cytoplasm (27). Mutations of the predicted surface area of the HSR domain cause the protein accumulation in the nucleus blocking its cytoplasmic localization probably enhancing nuclear import or inhibiting nuclear export (57). Mutations of the SAND domain disturb the distribution of Aire between the nucleus and cytoplasm suggesting a role for the SAND domain in nuclear transport mechanisms (57). Moreover, since the six helix CARD domain is involved in homodimerization, missense mutations in this region often affect Aire multimerization or localization to nuclear bodies (58) while most of the missense mutations in PHD domains alter the zinc-finger fold and decrease Aire’s transcriptional activation capacity (38).

Some different mutations have been found to be peculiar to certain populations. R257X is the most common mutation among Finnish and other European patients (59–61). R257X is a nonsense mutation, which most probably results in a carboxy-terminally truncated, non-functional Aire protein leading to alterated subcellular localization and inhibition of the transactivation function and complex formation of Aire (27). The 1094–1106 del113 (or 967–979 del-13 bp) is the most common mutation in British (62), Irish (63), North American (64, 65), and Norwegian patients (51) leading to the truncation and loss of function of Aire. Y85C is the only missense mutation found among Iranian Jews (57). In Italy, typical mutations of AIRE have been detected in Sardinia (R139X on exon 3) (50, 54), where this nonsense mutation leads to a total absence of Aire and seems to be associated with a more severe phenotype. In Apulia, the missense mutation W78R on exon 2, and the nonsense mutation Q358X on exon 9 have been found. The mutation Q358X lies in the PRR resulting in a truncated protein which lacks the second PHD finger and thereby is most likely non-functional protein (53). In Sicily, the most frequent mutation is R203X on exon 5, and two novel mutations, S107C and Q108fs on exon 3, have been detected. The mutation S107C is a missense mutation, whilst Q108fs is a small deletion, both affecting the HSR domain of Aire protein and it is likely that the Aire protein loses its homodimerization properties (66, 67). In Venetian patients, the most frequent mutations are R257X on exon 6 and 979 del-13 bp on exon 8, that are analogous to those detected in Finnish and Anglo-saxon patients but different from Italian ones (55). No typical mutations have been identified neither in Calabria nor in Campania (52, 68) even though patients from Campania show a high frequency of mutations in the exon/intron 1 junction. Compared to other mutations, the R257X results in a total loss of function, whereas the less dramatic truncations of the AIRE protein and many missense mutations, especially the predicted surface mutations of the HSR domain and the mutations in the leucine zipper domain, seem to exert less severe effects on the function of the Aire protein (27). Therefore, despite considerable variations in the APECED genotype, correlations with specific phenotypic features are far from being well elucidated. Only in patients affected with Candida infection, a correlation has been proved. In fact, candidiasis was significantly less prevalent in patients homozygous for 967–979del-13bp than in patients carrying the R257X or R139X, suggesting that AIRE truncation upstream the SAND domain promotes the susceptibility to this infection (69).

### Diagnosis of APECED

The onset of APECED usually occurs during childhood. The clinical diagnosis is based on the presence of two of the three classical components: chronic mucocutaneous candidiasis (CMC), chronic hypoparathyroidism (CH), and Addison’s disease (AD). The presence of only one of these features is sufficient for the diagnosis, when a sibling is affected. Molecular analysis of AIRE may help to confirm the clinical diagnosis, in particular in those cases with an atypical presentation (70, 71). Neutralizing autoantibodies against IFN-ω and IFN-α may represent a precocious biomarker detectable in the majority of patients and, thus they have been recently included in the diagnostic criteria of APECED (72).

### Clinical expression, autoantibodies profile, and susceptibility factors

APECED is characterized by a highly variable pattern of destructive autoimmune reaction, mainly mediated by specific autoantibodies toward different endocrine and non-endocrine organs. Virtually, all tissues and organs may represent the target of the autoimmune attacks, thus leading to a wide spectrum of clinical features. As already mentioned, the three main components of APECED are CMC, CH, and AD. CMC is, generally, the first component to develop, often followed by CH, before the age of 10 years and later by adrenal insufficiency (73, 74). In addition to the main components, the spectrum of minor manifestations may include ectodermal dystrophy, other endocrinopathies, such as hypergonadotripic hypogonadism, insulin-dependent diabetes, autoimmune thyroiditis, and pituitary dysfunction. Moreover, gastrointestinal disorders (chronic atrophic gastritis, pernicious anemia, malabsorption, autoimmune hepatitis and cholelithiasis), skin diseases (vitiligo and alopecia), keratoconjunctivitis, immunological defects, asplenia may be present (70). More rare manifestations of the disease include immune-mediated central and peripheral neurological manifestations, such as chronic inflammatory demyelinating polyneuropathy (54) and posterior reversible encephalopathy syndrome (PRES) (75), tubulointerstitial nephritis, autoimmune bronchiolitis, reversible metaphyseal dysplasia, hypokalemia, and hypertension (72).

The majority of APECED components have been correlated with specific autoantibodies that may represent an useful tool for the diagnosis and the follow-up of patients (Table 1). Autoantibodies’ profile may parallel clinical expression even though a strong correlation with the phenotype and the severity of the disease is not always present. Indeed, only some autoantibodies are highly predictive of specific organ’s failure, being detectable years before the onset of the overt clinical manifestations.

**Table 1:** **Clinical counter part of autoantibodies profile in APECED [modified by Capalbo et al. (73)]**.

Clinical features	Autoantibodies
CMC	Abs against IL-22, IL-17F, and myosin-9
**ENDOCRINE MANIFESTATIONS**	
HP	Abs against NALP5
AD	Abs against CYP21, CYP11A1, CYP17
Ovarian failure	Ab against CYP11A1, CYP17, and NALP5
Type 1 diabetes	Ab against IA-2 and insulin
Autoimmune thyroiditis	Ab against TPO and Tg
**NON-ENDOCRINE MANIFESTATIONS**	
Ectodermal manifestations
Vitiligo	Abs against Melanocytes, SOX-9, SOX-10, and AADC
Alopecia	Abs against TH
Gastrointestinal manifestations
Autoimmune gastritis/pernicious anemia	Abs against parietal cells and IF
Autoimmune hepatitis	Abs against CYP-1A2, CYP-2A6, AADC, and TPH
Autoimmune enteropathy	Abs against TPH, HD, and GAD
Rare manifestations
Pulmonary disease	Abs against KCNRG
Demyelinating polyneuropathy	Abs against myelin protein zero
Tubular interstitial nephritis	Abs against proximal tubule
Non-organ specific Abs	Abs against IFN-α and IFN-ω

*Abs, autoantibodies; IL-17F, interleukin 17F; IL-22, interleukin 22; NALP5, NACHT leucine-rich-repeat protein 5; CYP21, 21-hydroxylase; CYP11A1, cholesterol side-chain cleavage enzyme; CYP17, 17-α-hydroxylase; IA-2, tyrosine phosphatase-like protein; TPO, thyroid peroxidase; Tg, thyroglobulin; AADC, aromatic l-amino acid decarboxylase; TH, tyrosine hydroxylase; IF, intrinsic factor; TPH, tryptophan hydroxylase; HD, histidine decarboxylase; GAD, glutamic acid decarboxylase; CYP-1A2, cytochrome P450 1A2; CYP-2A6, cytochrome P450 2A6; KCNRG, potassium channel-regulating protein; IFN-α, interferon α; IFN-ω, interferon ω*.

APCED-related CMC has been associated with the presence of specific autoantibodies against the Th17-related cytokines interleukin- (IL-) 22 and IL-17F (76, 77). A parathyroid-specific autoantigen called NACHT leucine-rich-repeat protein 5 (NALP5), which is expressed in the cytoplasm of the main cell type in the parathyroid glands (78), has been recently proposed as the immunological hallmark of APECED-related CH.

Antibodies against the enzyme 21-hydroxylase (CYP21) are strongly associated and highly predictive for the development of AD in patients with CH and/or CMC (79, 80). Steroidogenic enzymes such as Cholesterol side-chain cleavage enzyme (CYP11A1) and 17α-hydroxylase/17,20-lyase (CYP17) represent a further targets of autoimmune reaction against adrenal cortex, moreover they are highly correlated with ovarian insufficiency due to lymphocytic oophoritis and can precede the clinical onset of the component (81, 82). Autoimmune gastritis is associated with the presence of autoantibodies against parietal cells and intrinsic factor (IF), the latter being involved in the development of pernicious anemia (83). The presence of autoantibodies against tryptophan hydroxylase (TPH), an enzyme involved in the synthesis of neurotransmitters in the nervous system and in the gastrointestinal endocrine cells correlates with Autoimmune enteropathy (84–87). Moreover, autoantibodies against both histidine decarboxylase (HD), an enzyme expressed in entero-chromaffin-like cells, and GAD (88, 89) have been associated with an autoimmune intestinal involvement. AH is mainly associated with the presence of autoantibodies against cytochrome P4501A2 (CYP-1A2), CYP-2A6, and aromatic l-amino acid decarboxylase (AADC), even though other types of autoantibodies, such as those directed against TPH, have been correlated with the AH component of the APECED phenotype (54, 90–92). Complement-fixing melanocyte autoantibodies and antibodies against transcription factors SOX-9, SOX-10, and AADC (83, 89) and tyrosine hydroxylase (TH) strongly correlate with the presence of vitiligo and alopecia (72, 83). Recently, several reports have confirmed an important role of autoantibodies against IFN-α and IFN-ω, which, although not tissue-specific, have been detected in the serum of almost all APECED patients (93, 94). Furthermore, they appear at a very early stage, often before the onset of any clinical manifestation. With this regard, their presence may be considered as an additional diagnostic marker of the disease, especially in those cases with an atypical presentation (94, 95). Although autoantibodies’ production seems to be a key-event in the development of the clinical disease, their role in the pathogenesis of APECED still remains to be defined.

APECED is a paradigmatic example of an autoimmune monogenic disease, however, the phenotypic presentation can widely vary from one patient to another (67, 70, 96, 97). Indeed, there are observations documenting a genotype-phenotype correlation only for specific traits (98, 99), but a clear genotype-phenotype correlation is lacking. We have, recently, reported on a family with an extremely wide intra-familial clinical variability despite the same mutation of AIRE (100). These observations suggest that genetic background is not able to explain alone the variability of the clinical expression and the severity of APECED and that, as for other monogenic diseases, the phenotypic variability of the syndrome may result from the complex interaction between several genetic, epigenetic, immunological, and/or environmental factors. The HLA class I and class II alleles have been reported to confer susceptibility to develop autoimmune diseases, such as Type 1 diabetes and autoimmune thyroid diseases (101). Only few studies investigated the association between the APECED phenotype and HLA genotypes, reporting conflicting results. In fact, although some studies did not find any significant association between HLA antigens class I or II and autoantibodies’ production or clinical expression of the disease (74, 102–104), other showed an increased frequency of specific HLA genotypes in APECED patients (105). However, in a more recent study on 18 Sardinian patients (54) autoimmune hepatitis, as well as LKM autoantibodies, have been found to be strongly associated with HLA-DRB1*0301/DQB1*0201. However, there is no evidence indicating that the HLA haplotype might be associated to a particular severity of the disease. Infectious agents are potent stimuli for the immune system, and thus both viruses and bacteria can be considered as trigger of an autoreaction via different mechanisms, such as molecular mimicry, bystander activation, and epitope spreading (106–111). Moreover, evidence suggests that a genetically determined susceptibility may favor the development of an autoimmune disorder after an infection. Many viruses have also been proposed as factors exacerbating several autoimmune processes (112). However, the role of the infectious triggers has not been sufficiently investigated in patients with APECED, and preliminary results did not show any significant effect of different infections on the phenotypic expression of the syndrome (100). As already mentioned, along with the central tolerance network, which is primarily involved in the pathogenesis of APECED, several peripheral mechanisms are capable of contributing to the control and regulation of the immune system. These factors are involved in maintenance of the homeostasis by controlling residual auto-reactive clones, which escape negative selection within the thymus and play a significant role in preventing or minimizing reactivity to self-antigens. The peripheral tolerance recognizes as possible mechanisms the induction of functional anergy with inactivation of self-reactive T-cells, deletion of auto-reactive clones by apoptosis, through Fas/FasL interaction, and the suppressive action of Tregs. An additional mechanism involved in controlling reactivity to self engages in the periphery is represented by NK cell activity. A possible role of altered peripheral tolerance in the pathogenesis and clinical expression of APECED might be hypothesized also considering that recent evidence suggesting that Aire may also be implicated in the control of peripheral mechanisms dedicated to the peripheral maintenance of self-tolerance. In the periphery, Aire is expressed in DCs and a specific population of extrathymic Aire-expressing cells (113, 114). As in the thymus, also in secondary lymphoid organs Aire is required for the expression of many TSAs. However, only few studies investigated the functionality of peripheral tolerance mechanisms in patients with APECED and the role of a failure in the peripheral mechanisms of Aire’s function is still poorly defined. Studies on animal models of APECED suggest that Aire does not influence *per se* Tregs as in Aire-KO mice the number of CD4+CD25+ cells are normal, and the functionality in *in vitro* suppression assays is normal as well (115, 116). However, the link between Aire and Treg cells is still not fully understood. Some recent studies suggest that Aire-expressing mTECs are involved in the generation of TSA-specific Foxp3+ Treg cells. A recent study supports this concept by showing that Aire-expressing mTECs, in addition to providing an antigen reservoir, also serve as APCs, thus enhancing the selection of Treg cells. The commitment of Tregs was shown to occur independently of Foxp3, and interaction of developing thymocytes with thymic stromal cells may drive the differentiation of a thymocyte subpopulation into the Treg cell lineage and, subsequently, trigger the expression of Foxp3 (117). Some adult APECED patients have lower proportion of Tregs (118), this finding being probably related to chronic infections, to the extent of autoimmune inflammation or therapy. Unfortunately, Tregs have been evaluated in only two children with APECED. Although in these children the number of Tregs was reduced in comparison to healthy controls, confirming the results obtained in adult patients, this reduction was not related to the severity of the disease, thus ruling out a potential role in modulating the clinical expression of the syndrome (100).

## Closing Remarks

Although APECED is a monogenic autoimmune disease, the great variability of the clinical expression and the absence of a clear genotype-phenotype correlation implies that, beyond AIRE mutations, other susceptibility factors such as immunological and environmental factors may be involved in the pathogenesis of the disease. The evidence of a role of an impairment of central and peripheral tolerance and of other susceptibility factors in the phenotypic variability of APECED is limited and needs to be further investigated. So far, the reason of such variability still remains obscure. Unraveling the open issues of the molecular basis of APECED, will be extremely useful in improving the diagnosis, management, and therapeutical strategies of this complex disease. As for other Mendelian diseases, total exome sequencing could be a good perspective to analyze other genetic variations and to identify potential disease-modifying genes involved in the clinical expressivity of organ-specific autoimmunity.

## Conflict of Interest Statement

The authors declare that the research was conducted in the absence of any commercial or financial relationships that could be construed as a potential conflict of interest.
